# Stable and Functional Cosmetic Creams Enriched with Grape Stem Extract: A Sustainable Skincare Strategy

**DOI:** 10.3390/antiox14070784

**Published:** 2025-06-25

**Authors:** Mónica Serra, Cláudia Botelho, Hugo Almeida, Ana Casas, José António Teixeira, Ana Novo Barros

**Affiliations:** 1 CEB—Centre of Biological Engineering, University of Minho, 4710-057 Braga, Portugal; quality4@mesosystem.com (M.S.); claudiabotelho@me.com (C.B.); jateixeira@deb.uminho.pt (J.A.T.); 2 Mesosystem Investigação & Investimentos by Spinpark, Barco, 4805-017 Guimarães, Portugal; cto@mesosystem.com (H.A.); ana@mesosystem.com (A.C.); 3 LABBELS—Associate Laboratory, 4710-057 Braga, Portugal; 4 Centre of Molecular and Environmental Biology (CBMA), Aquatic Research Network (ARNET), Associate Institute of Science and Innovation for Sustainability (IB-S), University of Minho, Campus de Gualtar, 4710-057 Braga, Portugal; 5 UCIBIO, Laboratory of Pharmaceutical Technology, Faculty of Pharmacy, University of Porto, 4051-401 Porto, Portugal; 6 Associate Laboratory i4HB Institute for Health and Bioeconomy, Faculty of Pharmacy, University of Porto, 4051-401 Porto, Portugal; 7 Centre for the Research and Technology of Argo-Environmental and Biological Sciences (CITAB), Institute for Innovation, Capacity Building and Sustainability of Agri-Food Production (Inov4Agro), University of Trás-os-Montes and Alto Douro (UTAD), Quinta de Prados, 5000-801 Vila Real, Portugal

**Keywords:** grape stem extract, phenolic compounds, antioxidant activity, antiaging, cosmetic formulation, cream stability, bioactive compounds, winemaking by-products, natural cosmetics

## Abstract

The growing demand for sustainable and effective cosmetic ingredients has prompted renewed interest in winemaking by-products. Among these, grape stem (GS) extract remains relatively underexplored despite its rich content of phenolic compounds distinct from those found in more commonly studied grape seeds or skins. This study validates the potential of GS extract as a novel bioactive component in cosmetic cream formulations. Rich in antioxidant, antiaging, and depigmenting compounds—such as resveratrol, catechins, and phenolic acids—GS extract was incorporated into creams at concentrations ranging from 0.33% to 6.25%. The formulations were evaluated for physicochemical characteristics, texture, rheological behaviour, and biological activity. The results demonstrated that GS extract enhanced total phenolic and flavonoid content, as well as viscosity, firmness, and antioxidant capacity—although not always in a concentration-dependent manner. All formulations maintained appropriate pH values and microbiological stability. Accelerated stability tests (40 °C, 75% RH, 3 months) identified the 0.83% to 1.64% concentration range as the most stable, preserving phenolic content, viscosity, and bioactivity. Higher extract levels, in contrast, led to reduced formulation stability, coalescence, and diminished antioxidant performance over time. Notably, GS-enriched creams exhibited significant elastase and tyrosinase inhibition, with lower concentrations maintaining antiaging potential throughout storage. These findings not only demonstrate that the incorporation of GS extract into a cosmetic base preserves its biological functionality but also reinforce the unique value of grape stems as an untapped resource for cosmetic innovation. Overall, the study advances current knowledge by establishing formulation parameters for a stable, effective, and sustainable cream based on grape stem extract. Further studies are recommended to optimize extract concentration and investigate encapsulation strategies for enhanced bioactive delivery and long-term stability.

## 1. Introduction

The cosmetic industry is a fast-evolving, innovation-driven sector that plays a pivotal role in personal care and wellness. In 2022, the European cosmetic and personal care market reached a valuation of EUR 88 billion, positioning itself as one of the most valuable and competitive sectors globally [1,2,3]. This growth has been largely fueled by increasing consumer demand for products that are not only effective but also aligned with environmental and ethical values. There is a rising emphasis on sustainability, transparency, and the use of naturally derived ingredients that are safe for human use and have minimal environmental impact [4,5].

In recent years, consumer awareness has intensified around the potential adverse effects of synthetic cosmetic ingredients, such as allergic contact dermatitis, irritant dermatitis, phototoxicity, and photoallergic reactions [6,7,8,9]. Simultaneously, there is growing concern over the ecological footprint of cosmetic manufacturing processes, including resource depletion, energy consumption, and the environmental persistence of chemical residues [10,11]. These concerns have prompted a paradigm shift across the industry, which has moved from conventional petrochemical-based ingredients toward more sustainable, plant-derived alternatives that deliver comparable efficacy while offering improved biocompatibility and ecological safety [6,7,8].

In this context, natural products rich in bioactive compounds—particularly phenolic substances—have gained significant attention for their multifunctional cosmetic benefits [12,13]. These compounds, commonly found in fruits, vegetables, and herbs, are known for their antioxidant, anti-inflammatory, antimicrobial, and antiaging properties [14,15,16,17,18,19]. Notably, polyphenols can scavenge free radicals, modulate enzymatic activities, and influence signalling pathways associated with skin homeostasis and repair. Their use is particularly valuable in formulations aimed at protecting the skin from oxidative stress, inflammation, and premature aging—factors exacerbated by pollution, UV exposure, and lifestyle habits [7,18,19,20,21].

While grape-derived ingredients such as seed or skin extracts have been extensively studied, grape stems—a lignocellulosic by-product of winemaking—remain comparatively underutilized. However, this fraction of the plant contains a unique profile of phenolic compounds, including resveratrol, catechins, quercetin, and phenolic acids, which differ both in concentration and biological behaviour from those found in seeds or pomace [14,15,16,17]. Recent studies, including that by Serra et al. [22] have shown that grape stem extract exhibits high antioxidant activity and depigmenting capacity, and is non-cytotoxic to skin cell lines, making it particularly promising for dermocosmetic applications.

Therefore, the valorisation of grape stems in cosmetic formulations not only offers a sustainable alternative to synthetic active agents but also addresses a critical gap in the literature concerning this specific by-product. This approach contributes to waste reduction and resource optimisation in the viticulture sector, in line with the European Green Deal and the EU Bioeconomy Strategy [17,21,23] and supports the development of high-value applications for agricultural by-products, strengthening the link between sustainability, innovation, and economic resilience [24,25].

Moreover, the safety and regulatory compliance of natural ingredients are paramount in cosmetic product development. The Cosmetic Ingredient Review (CIR) has already validated 24 grape-derived ingredients for topical use, reflecting the industry’s commitment to rigorously evaluating the safety and efficacy of natural substances [26]. The European Union enforces strict guidelines through Cosmetics Regulation (EC) No 1223/2009, which governs aspects ranging from ingredient toxicity and microbiological stability to labelling and manufacturing practices [27]. Adherence to Good Manufacturing Practices (GMP) under ISO 22716, as well as standardised stability assessment protocols (ISO/TR 18811 and Cosmetics Europe guidelines), ensures product quality, reproducibility, and consumer protection [28,29,30].

Given this background, the present study investigates the incorporation of phenolic extracts derived from grape stems into a water-based gel cream cosmetic formulation. Unlike previous studies centered on grape seeds or pomace, our work focuses specifically on the formulation behaviour, biological functionality, and long-term stability of grape stem extract in a topical matrix. The objective is to characterise the physicochemical performance and preliminary stability of the formulation while demonstrating the feasibility of utilising this scarcely valorised by-product in skincare. By bridging scientific innovation and sustainability, this research contributes to the growing body of knowledge on eco-conscious cosmetic development and highlights the untapped potential of grape stems as a source of high-value “actives” for topical applications.

### Regulatory and Safety Considerations

The incorporation of grape stem extract in cosmetic formulations aligns with the regulatory framework established by the European Union, particularly Regulation (EC) No 1223/2009, which governs the safety, efficacy, and labeling of cosmetic ingredients. The extract components must be listed under the International Nomenclature of Cosmetic Ingredients (INCI), ensuring transparency and standardization for consumer safety.

Previous studies have demonstrated the safety profiles of grape-derived ingredients, including grape stem extracts, with no reported cytotoxicity or skin irritation effects at relevant concentrations [22,26]. Dermatological compatibility tests have confirmed their suitability for topical application, supporting their use as bioactive ingredients with antioxidant and antiaging properties. Nonetheless, comprehensive toxicological assessments and preservative efficacy tests remain essential steps before any commercial product launch, as regulated by current legislation and cosmetic safety guidelines.

## 2. Materials and Methods

### 2.1. Chemicals and Reagents

Gallic acid (3,4,5-trihydroxybenzoic acid) and acetic acid (all extra pure > 99%) were obtained from Panreac Química S.L.U. (Barcelona, Spain). Sodium hydroxide (98%), sodium nitrite, aluminium chloride (purity levels > 99%), and ethanol were purchased from Merck (Darmstadt, Germany). Catechin (98%), TROLOX (6-hydroxy-2,5,7,8-tetramethylchroman-2-carboxylic acid, purity ≥ 98.0%), ABTS^•+^ (2,2′-azino-bis-(3-ethylbenzothiazoline-6-sulfonic acid) diammonium salt, purity ≥ 98.0%), potassium persulfate (purity ≥ 99.0%), TPTZ (2,4,6-Tripyridyl-s-Triazine, purity ≥ 98.0%), iron (III) chloride (purity ≥ 99.9%), tyrosinase and elastase enzymes, and all reagents used in enzymatic assays were sourced from Sigma Aldrich (Steinheim, Germany). Hydrochloric acid (≈37%) was obtained from Honeywell Fluka (Seelze, Germany). Potassium ferricyanide was purchased from Acros Organics (Geel, Belgium).

Polyacrylamide, C13-14 isoparaffin, and Laureth-7 were supplied by Química Massó (Barcelona, Spain). Methylchloroisothiazolinone and methylisothiazolinone were obtained from Escuder (Barcelona, Spain). The fragrance (parfum) was provided by Inarom (Barcelona, Spain). Distilled water was produced using a Millipore water purification system (Millipore, Bedford, MA, USA).

### 2.2. Incorporation of Phenolic Compounds in Cosmetic Formulation

The initial stage in incorporating the phenolic compounds into cosmetic formulations involved extracting these compounds from grape stems. Subsequently, a cosmetic formulation was prepared by incorporating the phenolic compounds through mixing.

#### 2.2.1. Preparation of Stem Extracts

The extraction of phenolic compounds from grape stems was based on the protocol described by Costa et al. [31], with minor adaptations. Briefly, 18 g of dried and ground grape stem material were mixed with 198 mL of a hydroalcoholic solution (ethanol/water, 70:30 *v*/*v*) and stirred for 30 min at 300 rpm. The mixture was then centrifuged (ScanSpeed 1580R, Labogene, Denmark) at 4000 rpm for 15 min. This process was repeated three times to maximize extraction efficiency. The combined supernatants were adjusted to a final volume of 500 mL using the same extraction solvent.

Ethanol was subsequently removed by rotary evaporation (Stuart RE300, Cole-Parmer, St. Neots, UK), and the aqueous phase was freeze-dried using a Benchtop Pro Freeze Dryer with Omnitronics™ (VirTis, SP Scientific, Warminster, PA, USA) to obtain the dry GS extract powder.

#### 2.2.2. Preparation of Cosmetic Formulations

A commercial water-based gel cream base (Química Massó, Barcelona, Spain) was used as the vehicle for all formulations. This base contains a standardized preservative system permitted under Annex V (Ref. No. 39) of Regulation (EC) No 1223/2009, but does not include any declared antioxidant or phenolic active ingredients. The control sample (CRT) consisted of the unmodified base cream and served as the reference formulation in all comparative evaluations. No ethanol was added to the control formulation; any traces present in the enriched samples were removed during rotary evaporation and freeze-drying of the grape stem extract. The GS extract powder was incorporated into 30 g of cream base using gentle mechanical stirring until fully homogenized. No heating was applied, to preserve the integrity of the thermolabile phenolic compounds. The final concentrations of extract in the formulations ranged from 0.33% to 6.25%, as shown in Table 1.

These concentrations were selected based on previous findings by Serra et al. [22], who evaluated the in vitro biological activity of GS extract at different levels and confirmed its safety and functionality on skin cell lines. The lower range was intended to explore minimal effective doses, while the higher concentrations allowed for assessment of stability and potential saturation or adverse effects on the formulation matrix.

The composition details of the water gel cream base can be found in Table 2.

It is important to note that, although this study evaluated formulation stability at two temperatures (4 °C and 25 °C), no photostability or oxidative exposure testing was performed. Light and oxygen are known to accelerate degradation of phenolic compounds and antioxidants. Therefore, future studies should include storage under light and air exposure conditions, to more accurately simulate consumer use and to assess the real-world oxidative stability of the cream matrix.

### 2.3. Cosmetic Formulation Characterization and Accelerated Stability Evaluation

Cosmetic formulations based on grape stem (GS) extract were characterized following ISO/TR 18811, the “Guidelines on the stability testing of cosmetic products” [29] The stability metrics assessed included organoleptic characteristics, pH, texture, and rheological properties. The formulation’s phenolic compounds were evaluated, including total phenolic and flavonoid contents. Antioxidant activity was assessed through ABTS and FRAP assays, while antiaging and depigmenting activities were determined using elastase and tyrosinase inhibition assays.

#### 2.3.1. Organoleptic Characteristics and pH Measurement

The organoleptic features, including colour, appearance, and odour, were subjected to visual evaluation.

The pH values were directly determined, at room temperature, using a pH meter (pH MesuLab PHS-3E).

#### 2.3.2. Evaluation of Texture and Rheological Properties

Texture parameters, including firmness and adhesiveness, were evaluated using a texturometer, specifically the Stable Micro Systems TA-HD plus (Haslemere, UK). The evaluation encompassed the following test conditions: compression mode, cylinder probe (10 mm, P/10), penetration distance of 5 mm, test speed of 3 mm/s, trigger force of 0.049 N, and a loading cell of 5000 g.

Rheological measurements were performed at 25 °C in a Discovery HR-1 rheometer equipped with a Peltier plate (TA Instruments, New Castle, DE, USA). Flow curves were obtained by using a 40 mm plate and performing a two-step program (up-down), using a continuous ramp and shear rate range between 0.1 and 500 s^−1^. The two-step program was carried out to access the thixotropy of the formulations.

#### 2.3.3. Biological Contamination Monitoring

Evaluation of biological contamination was made by spreading uniformly 1 g of each cream formulation, using a sterile swab, across the agar surfaces of two distinct media: Trypticase Soy Agar (TSA) for quantifying mesophilic aerobic bacteria and Sabouraud Dextrose Agar-Chloramphenicol (SDA-C) for quantifying yeast and fungi. Incubation of the plates took place at 30–35 °C for 5 days (TSA) and at 20–25 °C for 7 days (SDA-C) in the incubators (VWR). The results are presented in colony-forming units per gram of the sample (CFU/g).

#### 2.3.4. Extract Preparation from Creams

To assess the phenolic content and the antioxidant, antiaging, and depigmenting activities of the cosmetic formulation containing grape stem extract (CF-GS), the phenolic compounds, which were the bioactive ingredients in the formulation, were extracted from the cream base. After the extraction process, both the phenolic contents and the biological activities were determined.

The preparation of the cream extract followed the procedure delineated by Mapoung et al. [32], with certain modifications. The process began by weighing 1 g of cream, followed by freezing it at −20 °C for 15 to 20 min. Subsequently, 9 mL of ethanol was added, and the mixture suffered vortexing and centrifugation at 15,000 rpm for 15 min. The resulting supernatant was saved, and subsequent dilutions were prepared prior to conducting the tests.

#### 2.3.5. Quantification of Phenolic Compounds

The quantification of phenolic content in CF-GS utilized modified spectrophotometric methods specifically designed for 96-well microplates (PrimeSurface MS-9096MZ, Frilabo, Maia, Portugal), as outlined by Costa et al. [31]. Absorbances were subsequently determined using microplate readers (Multiskan GO Microplate Photometer, Thermo Fisher Scientific, Vantaa, Finland).

##### Determination of Total Phenolic Content

The determination of the total phenolic content (TPC) in CF-GS was conducted using the Prussian blue assay, a method involving the formation of a blue-coloured complex resulting from a reaction between different components. The procedure started with the addition of 100 µL of a 0.5 mmol/L ferric chloride hexahydrate solution and 100 µL of a diluted sample to a microplate well, where they were allowed to react for 2 min. Following this, 100 µL of a 0.5 mmol/L potassium ferricyanide solution was introduced. After shaking the microplate for 20 s, the absorbance was measured at 725 nm after 15 min. To construct the analytical curve, gallic acid was utilized at various concentrations. The total phenolic content was quantified and expressed as milligrams of gallic acid equivalent per 100 g of the sample (mg GAE/100 g sample).

##### Determination of Flavonoid Contents

The methodology utilized to determine flavonoid content (FC) involved the creation of a flavonoid–aluminium complex. In this procedure, 24 µL of the diluted sample was introduced into a microplate well, followed by the addition of 28 µL of a 50 g/L sodium nitrite solution. After a 5 min reaction period, 28 µL of a 100 g/L aluminium chloride solution was added. Following a 6 min reaction period, 120 µL of a 1M sodium hydroxide solution was introduced into the well. The microplate was then stirred for 30 s, and the absorbance was measured at 510 nm. For the construction of the calibration curve, various concentrations of catechin were employed. The results are expressed as milligrams of catechin per 100 g of sample (mg CAT/100 g sample).

#### 2.3.6. Antioxidant, Antiaging, and Depigmenting Activities

Biological activities were assessed using adapted spectrophotometric techniques designed for 96-well microplates (PrimeSurface MS-9096MZ, Frilabo, Maia, Portugal), following the protocols described by Costa et al. [31] and Taghouti et al. [33]. Absorbance readings were then recorded using microplate readers (Multiskan GO Microplate Photometer, Thermo Fisher Scientific, Vantaa, Finland).

##### ABTS Assay

The initial step in this assay involved the oxidation of the ABTS salt, achieved by introducing a 148 mM potassium persulfate solution (88 µL) to a 7 mM ABTS solution (5 mL). The mixture was allowed to rest for 12 to 16 h at room temperature, protected from light, to attain its most stable oxidative state.

Subsequently, the ABTS^•+^ working solution was prepared by diluting the cationic ABTS^•+^ solution in a 20 mM sodium acetate buffer (pH 4.5) until an absorbance of 0.700 ± 0.020 at 734 nm was achieved.

Following this, the evaluation of the sample’s antioxidant capacity involved adding 188 µL of the ABTS^•+^ working solution to each microplate well, followed by the introduction of 12 µL of the diluted sample and blank. After a 30 min incubation period at room temperature, shielded from light, the absorbance was measured at 734 nm [Abs (734 nm)]. TROLOX at various concentrations served as the standard for constructing the analytical curve, with water acting as the blank.

The ABTS assay estimated the scavenging capacity of ABTS^•+^ by the samples, and the inhibition percentage was calculated using the following formula:

$${\%\;\mathrm{inhibition}}_{\mathrm{Sample}}=\frac{{\mathrm{Abs}\;(734\;\mathrm{nm})}_{\mathrm{Blank}}-{\mathrm{Abs}\;(734\;\mathrm{nm})}_{\mathrm{Sample}}}{{\mathrm{Abs}\;(734\;\mathrm{nm})}_{\mathrm{Blank}}}\;\times \;100$$

The results are presented in TROLOX Equivalent Antioxidant Capacity (TEAC) and expressed in millimoles of TROLOX per 100 g of sample (mmol TROLOX/100 g sample).

##### FRAP Assay

The FRAP method for assessing antioxidant activity is initiated by the preparation of the FRAP working solution. This involved combining 10 volumes of 220 mM acetate buffer (pH 3.6), 1 volume of a 40 mM TPTZ (2,4,6-Tripyridyl-s-Triazine) solution dissolved in 40 mM HCl, and 1 volume of 20 mM ferric chloride. The freshly prepared FRAP working solution was preheated to 37 °C 10 min before use.

Following this preparation, 20 µL of the sample was added to a microplate well, followed by the introduction of 280 µL of the preheated FRAP working solution. The microplate was then shaken and placed in darkness, incubating at 37 °C for 30 min, after which the absorbance was measured at 593 nm. The calibration curve was constructed using various concentrations of TROLOX. The reported results were in millimoles of TROLOX per 100 g of sample (mmol TROLOX/100 g DW).

##### Tyrosinase Inhibition Assay

The tyrosinase inhibition assay measured the percentage of tyrosinase inhibition, following a specified procedure. Initially, 20 µL of 1000 U/mL tyrosinase and 170 µL of a mixture (containing 1 mM L-tyrosine solution, 50 mM phosphate buffer at pH 6.5, and water in a 10:10:9 ratio) were added to the sample. Subsequently, the microplate experienced incubation at 37 °C for 10 min, and the absorbance was read at 490 nm. Positive and negative controls, using 1 mg/mL Kojic acid and 10% DMSO, respectively, were included. The percentage of inhibition was calculated using the following formula:

$${\%\;\mathrm{inhibition}}_{\mathrm{Sample}\;}=\frac{{\mathrm{Abs}\;(490\;\mathrm{nm})}_{\mathrm{Negative}\;\mathrm{Control}}-{\mathrm{Abs}\;(490\;\mathrm{nm})}_{\mathrm{Sample}}}{{\mathrm{Abs}\;(490\;\mathrm{nm})}_{\mathrm{Negative}\;\mathrm{Control}}}\;\times \;100$$

##### Elastase Inhibition Assay

The elastase inhibition assay was designed to determine the percentage of elastase inhibition. In this assay, 50 µL of the sample was combined with 160 µL of 0.20 mM Tris-HCl buffer (pH 8) and 20 µL of 0.80 mM N-Succinyl-Ala-Ala-Ala-p-nitroanilide substrate (in Tris-HCl buffer). After a 10 min incubation at room temperature, 20 µL of 1 U/L elastase enzyme (in Tris-HCl buffer) was added, and the microplate underwent an additional 20 min incubation at room temperature. The absorbance was then measured at 410 nm, with Tris-HCl buffer serving as the negative control. The percentage of inhibition was calculated using the following formula:

$${\%\;\mathrm{inhibition}}_{\mathrm{Sample}}=\frac{{\mathrm{Abs}\;(410\;\mathrm{nm})}_{\mathrm{Negative}\;\mathrm{Control}}-{\mathrm{Abs}\;(410\;\mathrm{nm})}_{\mathrm{Sample}}}{{\mathrm{Abs}\;(410\;\mathrm{nm})}_{\mathrm{Negative}\;\mathrm{Control}}}\;\times \;100$$

### 2.4. Accelerated Stability Study

The cream’s physicochemical stability was assessed through accelerated stability testing, involving storage at an elevated temperature (40 ± 2 °C) and relative humidity (75 ± 5%) for 3 months within a climatic chamber (Shjianheng SDJ-150GSD, Jianheng Instrument Co., Shanghai, China). The analysis included organoleptic characteristics, pH, rheological behaviour, and total microbiological contamination, as well as parameters related to phenolic and flavonoid content, and antioxidant, antiaging, and depigmenting activities. This comprehensive evaluation aimed to elucidate the cream’s stability under accelerated conditions, as well as its temporal performance.

In the context of accelerated stability testing, the Q rule (Q10 rule) is commonly employed to estimate the rate of reaction changes with temperature fluctuations. Within the cosmetic industry’s Accelerated Stability Model, the conservative assumption of Q = 2 is often adopted, suggesting that the reaction rate doubles for every 10 °C increase in storage temperature [34]. This cautious approach was implemented to provide a prudent estimate of the impact of temperature variations on the reaction rate in cosmetic formulations, allowing for a careful prediction of potential degradation or alterations in the cosmetic product over time.

Consequently, Table 3 illustrates the correspondence, based on the Q rule (Q = 2), between the storage times of samples under accelerated stability conditions and the storage times under normal conditions.

### 2.5. Microbiological Analysis

This study followed the ISO 17516:2014 guidelines in assessing the microbiological stability of the formulations under accelerated storage conditions. The preservative system used in the cream base includes substances listed under Annex V, Ref. No. 39 of Regulation (EC) No 1223/2009. In this preliminary phase, the preservative concentration remained within the legally permitted limits and was applied under controlled laboratory conditions solely for formulation stability evaluation.

As this is an initial development study, preservative efficacy testing (challenge test, ISO 11930 [35]) was not conducted. However, such testing will be mandatory and rigorously implemented in future stages prior to any regulatory submission or potential commercialization, in order to ensure full compliance with EU cosmetic legislation and product safety requirements.

### 2.6. Statistical Analysis

The data were analysed using IBM SPSS 29.0 statistical software through analysis of variance (ANOVA) and a multiple range test (Tukey’s test) for a significance level set at *p* < 0.05.

The results are expressed as mean values ± standard deviation and the tests were conducted in triplicate (*n* = 3).

## 3. Results and Discussion

### 3.1. Physical–Chemical Characterization and Accelerated Stability Evaluation of the New Cosmetic Formulation

Phenolic compounds extracted from grape stems were incorporated into a cream base, resulting in a cosmetic formulation. Distinct concentrations of the bioactive compounds were incorporated into the cream base, resulting in different formulations, which were thoroughly characterised following the guidelines outlined in ISO/TR 18811 “Cosmetics Europe: Guidelines on the stability testing of cosmetic products”. This comprehensive assessment encompassed the evaluation of various organoleptic properties, including colour, appearance, and odour, as well as the measurement of pH and viscosity [29,30]. Firmness, adhesiveness, and thixotropy were also assessed.

It was hypothesized that the incorporation of phenolic compounds would confer important characteristics upon the formulation, such as antioxidant, antiaging, and depigmentation activity; therefore, these parameters, including the quantification of the phenolics and flavonoids, were analysed.

The final assessment focused on stability, as it is known to be crucial for product safety, effectiveness, and quality. The assessment of stability focuses on colour, texture, composition, and active-ingredient concentration changes over-time. This evaluation is mandatory, as described in the EU Cosmetic Regulation, before entering the market [27]. This regulation highlights long-term stability assessment, but it lacks specific protocols for data acquisition. Therefore, this study focused on the guidelines provided by ISO 18811:2018 [29] and Cosmetics Europe [30]. These documents recommended that the formulations should be tested under elevated temperature and humidity, using the Q rule to estimate durations of shelf life. In alignment with the recommendations, the stability of the developed formulations was assessed under storage at a high temperature, 40 °C, and humidity, 75%, for a period of three months, which is equivalent to 12 months under normal conditions.

#### 3.1.1. Evaluation of Organoleptic Features and Their Stability

The evaluation of organoleptic properties in cosmetic formulations is essential for product differentiation and quality assurance. Changes in these parameters may indicate chemical alterations or microbiological contamination [3]. Table 4 details the organoleptic characteristics of the formulation immediately after its preparation (time 0). The cream base exhibited a bright “whiteish” colour, gradually changing to browner tones with the incorporation of higher concentrations of the bioactive molecules.

Fiume et al. [36] classified grape-derived ingredients as colourants, a determination which is corroborated in this study, as the incorporation of the GS extract concentration had a direct impact on the formulations’ visual attributes, particularly colour. Similar colour changes were reported by Hübner et al. [37] and Kawarkhe et al. [38]; in these studies, the inclusion of grape by-product led to the appearance of purplish or brown tones.

Texture changes were also observed; while the incorporation of low concentrations of extract maintained a creamy, soft texture, those with higher extract content (≥0.99%) exhibited a thinner consistency. The incorporation of extract may disrupt the internal emulsion structure. Supriadi et al. [39] similarly noted that a formulation with 5% grape seed oil displayed a gentle texture, suggesting an impact of the extract on the formulation rheology. To validate this hypothesis, specific rheological studies were performed.

#### 3.1.2. Texture and Rheological Properties

The base cream (control) exhibited a characteristic perfume scent. However, as the concentration of GS extract increased in the formulation, a woody aroma became progressively more prominent. Notably, formulations containing 3.85% and 6.25% GS extract displayed a distinctly woody fragrance that overshadowed the original perfume scent.

Ruiz-Moreno et al. [40] identified a diverse range of odour profiles in GS extract, including woody, almond, sweet, fruity, and caramel notes. Similarly, Hübner et al. [37] reported a characteristic grape-derived scent in preparations containing grape pomace. It is important to note that odour perception is inherently subjective and influenced by individual preferences; while some users may appreciate the woody, natural aroma, others may favour the original perfume fragrance. During the stability study, perceptible shifts in odour profiles were observed over time.

As mentioned, stability is an important feature in the development of a cosmetic formulation; therefore, the formulations were placed in a stability changer and evaluated over time regarding their visual changes in terms of colour.

In Table 5 it is clear that the colour changes over the period of three months. Notably, a marked intensification of colour was observed in formulations containing ≥3.23% GS extract.

The results obtained are consistent with those reported by Cefali et al. [41], who observed that formulations containing Benitaka grape peel extract exhibited colour variation by the 13th day of storage, highlighting their susceptibility to environmental factors. Similarly, Yarovaya et al. [42] described comparable colour changes in formulations containing 3% grape seed extract during stability studies. Over a three-month period at 30 °C, the colour shifted from light brown to brown, while storage at 50 °C accelerated the change to a darker brown. These alterations were attributed to the degradation of catechins.

The observed colour variation appears to correlate with the concentration of the extract and may be linked to chemical destabilization processes such as oxidation, hydrolysis, transesterification, and interactions among formulation components. These processes can degrade compounds and break chemical bonds, leading to the formation of new chemical species that contribute to the colour changes [32].

In terms of appearance, formulations containing up to 0.83% grape seed (GS) extract retained their texture throughout the storage period. Increasing the concentration to 0.99% maintained the texture for up to two months. However, higher concentrations (1.64% and 3.23%) preserved a soft texture only during the first month, after which the formulations exhibited increased lumpiness. These more concentrated formulations became progressively lumpier and more viscous after one month of storage in a stability chamber.

These findings align with those of Hernani et al. [43], who reported that formulations with higher concentrations (2–3%) of Gambier leaf extract became coarser after six weeks under accelerated conditions. Cefali et al. [41] also noted a slight increase in viscosity in grape peel extract-based formulations stored in an oven. The increased lumpiness observed in more concentrated samples can be attributed to the complex composition of the extract interacting with the cream base, which leads to particle or droplet coalescence. Coalescence, an irreversible process driven by thermal or mechanical energy, can significantly affect the texture, appearance, and viscosity of cosmetic formulations [44]

Throughout the stability study, all samples retained their odour. A transition from a parfum fragrance to a woody scent was observed in proportion to the GS extract concentration. This is consistent with the findings of Rodrigues et al. [45] and Hernani et al. [43], who also reported no significant odour alterations in similar formulations.

In conclusion, the concentration of GS extract in cosmetic formulations significantly influences colour, texture, and odour. While colour changes are noticeable, they are acceptable and do not imply structural instability, as they result from the natural characteristics of the GS extract. Similarly, olfactory transitions from parfum to woody tones are consistent with grape extract properties. Overall, these findings contribute to a deeper understanding of the impact of GS extract on the sensory and physical attributes of cosmetic products.

#### 3.1.3. pH Measurement and Microbiological Monitorization During Stability Evaluation

The pH of cosmetic formulations is a critical parameter that significantly influences both product stability and the product’s interaction with the skin. As an indicator of a solution’s acidity or alkalinity, pH plays a pivotal role in determining overall formulation performance, skin compatibility, and user experience. Therefore, precise pH evaluation is essential to ensure that cosmetic products meet the required specifications for efficacy and safety [46,47].

In this study, the pH values of the developed formulations were assessed. A clear trend of decreasing pH was observed with increasing concentrations of grape stem (GS) extract at time zero. Specifically, the pH decreased from 6.34 to 3.93 as the GS extract concentration increased. This increase in acidity is attributed to the phenolic composition of the extract, particularly the presence of proanthocyanidin trimer monogallate isomers, which are known to contribute to acidic properties.

Table 6 illustrates the pH behaviour of the samples during storage under accelerated conditions. Notably, there were no significant changes in pH over time, as the observed variations were minimal and remained close to zero. Across the storage period, the pH values of the formulations consistently ranged between 5.99 and 3.84, in accordance with the incorporated GS extract concentration (0–6.25%). These findings indicate that the formulations maintained pH stability throughout the storage period, supporting their physicochemical robustness.

As evident from the data, no significant differences in pH were observed over time, with variations remaining minimal. The formulations consistently maintained pH values between 5.99 and 3.84, corresponding to the GS extract concentrations ranging from 0% to 6.25%.

These findings are consistent with previously reported studies. Salem et al. [48], for example, formulated cosmetics containing 0–5% grape seed extract derived from the Marselan variety and observed a slight pH decrease from 5.33 to 5.26 as the extract concentration increased. Notably, the pH values of these formulations remained stable at approximately 5.3 over a four-month storage period. Similarly, Rodrigues et al. [49] reported a pH reduction from 9.65 to 9.39 upon the incorporation of 5% olive pomace extract, another phenolic-rich ingredient, into a facial mask. Supporting evidence was also provided by Gomes et al. [50] and Nešić et al. [51], who observed pH reductions from 6.04 to 4.11 with kiwi peel extract and from 6.61 to 5.69 with wild apple fruit extract, respectively.

Further reinforcing these trends, Rafique et al. [52] and Ferreira et al. [53] demonstrated that emulsions containing grape extracts consistently maintained pH levels within the acceptable range for skin compatibility during accelerated stability testing. In our study, the observed pH decreases from 6.34 (cream base) to values between 4.50 and 3.93 reflects an adjustment toward the skin’s natural acidity, which typically ranges between pH 4.0 and 4.5. This alignment with physiological skin pH is advantageous, as it supports the skin’s microbiota, preserves the integrity of the stratum corneum, and contributes to the maintenance of the barrier function and overall skin health [47]. As a result, the developed formulations are expected to exhibit good skin compatibility and effectiveness.

In terms of microbiological safety, all formulations remained within acceptable limits throughout the study, with microbial counts consistently below 100 CFU/g [54]. These results are in line with Rodrigues et al. [45], who found no microbial contamination in natural extract-based cosmetic formulations after 180 days of accelerated storage. Similar outcomes were reported by Gomes et al. [50], confirming the microbiological stability of such formulations.

In summary, the developed formulations demonstrated excellent pH stability and microbiological safety under accelerated storage conditions. These findings reinforce the formulations’ suitability for cosmetic use and their compatibility with skin physiology.

#### 3.1.4. Rheological Stability and Thixotropy Evaluation

##### Rheological Stability

An ideal Newtonian fluid is characterized by a linear relationship between shear stress and shear rate, with viscosity remaining constant regardless of applied force. However, most cosmetic formulations exhibit non-Newtonian behaviour, deviating from this ideal model [53,54,55]. To evaluate the rheological characteristics of the developed formulations, flow curves plotting shear stress or viscosity against shear rate were analysed

All formulations displayed pseudoplastic (shear-thinning) behaviour, which is common in cosmetic emulsions. In such systems, viscosity decreases as shear rate increases, facilitating ease of application and spreadability [45]

To quantitatively characterize this behaviour, the power-law model was applied:

σ = K·γnσ = K·γ^nσ = K·γn

where:

σ is the shear stress,

γ is the shear rate,

K is the flow consistency index,

n is the flow-behaviour index.

For shear-thinning fluids, n < 1; for Newtonian fluids, n = 1; and for dilatant fluids, n > 1. Lower n values correspond to shear-thinning properties which are more pronounced [56,57,58,59,60,61].

Application of the power-law model to the downward flow curves (to minimize structural breakdown artifacts) confirmed that all samples exhibited pseudoplastic behaviour, with nnn values below 1. The most intense shear-thinning was observed in the most concentrated sample (6.25% GS extract), with n = 0.1858, followed closely by the control (0% GS extract; n = 0.1878). Samples II and V (0.83% and 3.23% GS extract, respectively) showed the least shear-thinning behaviour, with nnn values of 0.2603 and 0.2365.

The flow consistency index (K), representing the force required to initiate flow, ranged from 46.697 to 161.19. The lowest K was found in the control (K = 46.697), while the highest was in sample III (0.99% GS extract, K = 161.19). Notably, K values did not strictly correlate with GS extract concentration, highlighting the influence of other formulation components and interactions.

These values align with the rheological properties observed in commercial cosmetic products such as sunscreen lotions (K ≈ 75, *n* ≈ 0.28) and mascara (K ≈ 200, *n* ≈ 0.24) [46]. Danilă et al. [56], using the power-law model on antiaging creams, reported n values between 0.508 and 0.606 and K values from 4.414 to 44.794, suggesting that lower consistency indices result in emulsions which are more fluid.

Additionally, Petkova-Parlapanska et al. [57] demonstrated that both the consistency and flow-behaviour indices vary depending on the cream base and extract composition. In one study using rosemary and grapefruit extracts, increasing the consistency index from 3.325 to 15.465 resulted in a reduction in the flow-behaviour index from 0.496 to 0.273, an effect reversed in another formulation with a different base. These variations were attributed to formulation-dependent factors such as pH, viscosity-donemodifying agents, and emulsifier contents.

**Figure 1:**
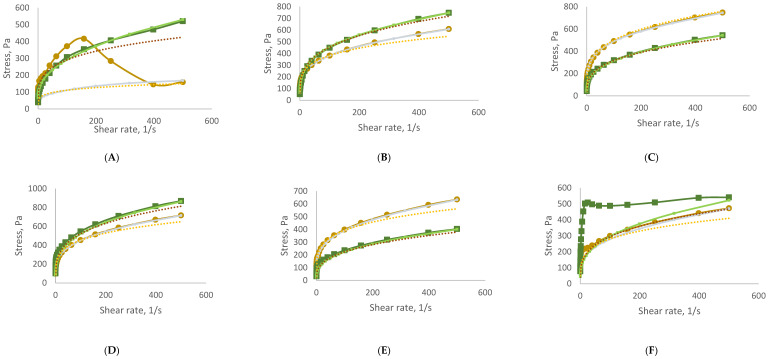

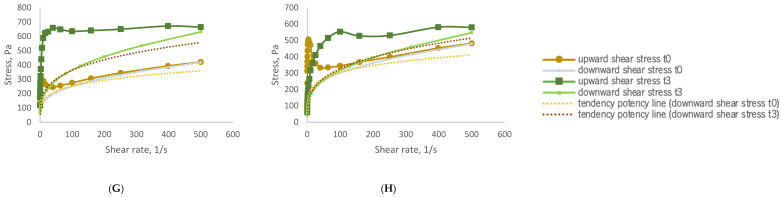
Rheograms of developed samples before (time 0) and at the end (time 3) of storage under accelerated conditions. (**A**)—sample CRT (0% GS extract); (**B**)—sample I (0.33% GS extract); (**C**)—sample II (0.83% GS extract); (**D**)—sample III (0.99% GS extract); (**E**)—sample IV (1.64% GS extract); (**F**)—sample V (3.23% GS extract); (**G**)—sample VI (3.85% GS extract); (**H**)—sample VII (6.25% GS extract). Rheological behaviour of formulations containing grape stem (GS) extract at time 0 (T0) and after accelerated storage (T3).

To assess rheological stability, all formulations were stored under accelerated conditions for three months. The flow curves obtained at the end of the storage period (time 3), as shown in Figure 1, continued to exhibit pseudoplastic behaviour. Each curve shows the relationship between shear stress and shear rate, reflecting the viscosity and flow behaviour of the samples. All formulations exhibit the non-Newtonian, shear-thinning behaviour typical of cosmetic gels and creams—meaning, viscosity decreases with increasing shear rate (the material becomes easier to spread). The addition of GS extract influences the internal structure, as seen in the variation in curve slopes across samples B–H. In general, higher GS concentrations led to increased initial viscosity and altered flow resistance. Over time (T3), most formulations maintained their shear-thinning character, suggesting good rheological stability, although minor shifts in curve profiles indicate subtle structural changes depending on GS content.

Reapplication of the power-law model to the time 3 data confirmed that all samples retained shear-thinning characteristics, with n values remaining between 0 and 1. However, both n and K values showed variations between time 0 and time 3, reflecting changes in the internal structure of the formulations during storage. These results are summarized in Table 7, demonstrating the formulations’ ability to maintain desirable rheological profiles over time.

##### Thixotropy

Thixotropy, another parameter that can be characterized in a rheogram analysis, describes the phenomenon in which the viscosity decreases over time when a sample at rest experiences flow, and then the viscosity recovers when the flow discontinues [62]. This parameter provides insights into how the cosmetic formulation returns to its original structure after the applied stress is removed. Moreover, topical formulations with thixotropic properties are desirable, as they become more fluid during the application, facilitating the spreading, and the recuperating of their initial characteristics, while preventing the product from running off the skin [56,57,58,59,60,61].

Thixotropy can be assessed by examining the hysteresis area in the rheograms, which represents the region between the upward and downward curves. A larger hysteresis area is indicative of a more pronounced time-dependent behaviour, which is typical of thixotropic materials. Consequently, such materials exhibit more significant variations in viscosity over time during both the flow and recovery phases [56,57,58,59,60]. When both curves coincide, it indicates the absence of thixotropy [58].

Figure 1 illustrates the thixotropic behaviour of the samples. Thixotropic behaviour correlates with the concentration of GS extract in the samples. Those with GS extract concentration levels between 0.33% and 1.64% display relatively low thixotropy, with hysteresis values ranging from 777 to 3086 Pa/s. In contrast, samples V (3.23% GS extract), VI (3.85% GS extract), and VII (6.25% GS extract) exhibit thixotropic behaviour which is more pronounced, as indicated by their significantly larger hysteresis area values of 8163, 10,931, and 17,487 Pa/s, respectively. This suggests an enhanced capacity for structural recovery.

Remarkably, the control sample demonstrates the highest capacity for structural recovery, with a hysteresis area of 62,121 Pa/s.

Similarly to this study, in which the thixotropy increased in more concentrated samples, Yarovaya et al. [63] investigated the thixotropy of samples containing 3% grape seed extract and observed an increase in thixotropy (from 1868 ± 32 to 3244 ± 78 Pa/s) with the addition of the extract to the sample. Pinto et al. [64] also examined the thixotropic behaviour of formulations containing Castanea sativa shells extract. They observed a 15% increase in thixotropy, compared to the control sample.

Within the study of stability, the rheograms provide compelling evidence of the evolving thixotropic behaviour of the samples. Both the control sample and sample I (0.33% GS extract) exhibit a loss of thixotropic capacity, as indicated by the nearly overlapping upward and downward curves. Conversely, the remaining samples (samples II, III, IV, V, VI, and VII) demonstrate an increase in hysteresis area in the rheograms, suggesting an enhancement in their thixotropic behaviour. This enhancement is particularly pronounced in samples III (from 2507 to 10,673 Pa/s), V (from 8163 to 62,725 Pa/s), VI (from 10,931 to 90,501 Pa/s), and VII (from 17,487 to 64,352 Pa/s). This enables these samples to return to their initial structure after stress application, highlighting their time-dependent behaviour.

Moraes et al. [62] examined the stability of formulations containing 0.4% rutin succinate. They evaluated thixotropy after storage in accelerated conditions for 90 days and noted a 38.32% increase in the hysteresis area, a finding consistent with our study. Specifically, the thixotropy of sample II (0.83% GS extract) increased by 46%.

##### Viscosity

Viscosity is a key rheological parameter that reflects a fluid’s resistance to flow or deformation, offering valuable insights into the structural integrity and application performance of cosmetic formulations. In this study, viscosity was evaluated at a shear rate of 500 s^−1^, which falls within the range typically encountered during the topical application of cosmetic products (100–10,000 s^−1^). This shear rate also represents the highest applied value in the experimental setup [65].

**Figure 2:**
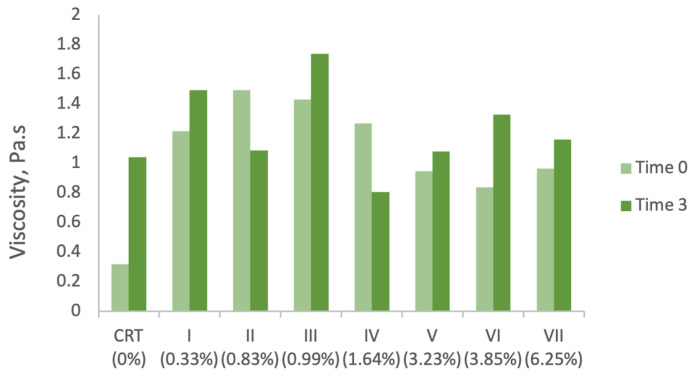
Viscosity variations in the developed formulations, measured at a constant shear rate of 500 s^−1^. The change in viscosity observed over the storage period under accelerated conditions did not follow a consistent or predictable trend. In general, most samples exhibited an increase in viscosity from the initial time point (before storage) to the final time point (after three months of storage). However, samples II (0.83% GS extract) and IV (1.64% GS extract) deviated from this trend, showing a decrease in viscosity. Importantly, these changes did not correlate linearly with the concentration of GS extract, suggesting that viscosity variation was influenced by other factors beyond extract content.

As shown in Figure 2, the incorporation of grape stem (GS) extract into the control sample initially led to an increase in viscosity, with the peak value recorded at a concentration of 0.83% GS extract (1.49532 Pa·s). Beyond this concentration, viscosity values decreased as GS extract levels increased further. This non-linear trend suggests that interactions between extract constituents and the emulsion matrix, as well as possible dilution effects or structural disruptions, may influence the formulation’s viscosity profile.

Comparable observations have been reported in the literature. For instance, Nunes et al. [66] noted similar viscosity behaviour upon incorporating 5% olive oil by-product extracts into cosmetic emulsions, ingredients also rich in phenolic compounds. Likewise, Yarovaya et al. [63] documented a viscosity increase (from 0.44 to 0.66 Pa·s at 200 s^−1^) upon adding 3% grape seed extract. García-Villegas et al. [67] also demonstrated that the viscosity of formulations increased proportionally with the concentration of *Mangifera indica* L. peel extract.

Viscosity changes were also monitored over a three-month accelerated storage period (time 0 vs. time 3). The results, presented in Figure 2, show that most formulations experienced a general increase in viscosity under these conditions. However, exceptions were observed in samples II (0.83% GS extract) and IV (1.64%), both of which exhibited a decrease in viscosity. The graph highlights the effect of grape stem (GS) extract concentration on viscosity over time, under accelerated storage conditions. A general trend of viscosity stability or slight decrease is observed, depending on the extract content. Formulations with higher GS concentrations tend to retain or enhance viscosity, suggesting the potential structuring effects of the bioactive compounds.

Among all samples, sample V (3.23%) demonstrated the least variation, with only a 14% increase in viscosity over time. Samples I (0.33%), III (0.99%), and VII (6.25%) also showed moderate increases, ranging from 20% to 23%. In contrast, sample IV showed the most substantial variation, with a 58% reduction in viscosity, suggesting greater structural instability during storage.

These results indicate that viscosity is influenced not only by GS extract concentration but also by storage-induced changes, potentially including coalescence phenomena, which were visually confirmed in several samples. Coalescence, in which smaller droplets or particles merge to form larger aggregates, can significantly affect the internal structure of emulsions, leading to viscosity fluctuations.

One likely explanation for this variability is the presence of visible lumps in several samples following storage. As previously discussed, one common physical destabilization mechanism in emulsified cosmetic products is coalescence, in which smaller dispersed droplets or particles merge to form larger aggregates. This process can lead to a heterogeneous texture and altered flow resistance, ultimately impacting viscosity. Both visual inspection and rheological analysis support the hypothesis that coalescence likely occurred in these formulations during storage.

The literature reports similarly diverse findings regarding viscosity stability in natural-extract-based cosmetic formulations. For example, García-Villegas et al. [67] observed a decrease in viscosity of up to 1.9 Pa·s in formulations containing 0.5%, 1%, and 2% *Mangifera indica* L. peel extract under accelerated storage. In the present study, the greatest viscosity variation (0.72 Pa·s) was found in the control sample (0% GS extract), reinforcing the finding that even base formulations without actives are subject to structural evolution.

Other studies have reported comparable effects. Anggraini et al. [68] noted viscosity decreases of 0.66%, 1.33%, and 0.89% in formulations with 0%, 5%, and 10% macassar kernel (*Rhus javanica*) stem extract, respectively, after 12 weeks of storage. Mahmood et al. [69] described a more pronounced reduction in viscosity in green tea extract formulations, from 18.01 cP to 2.62 cP after 30 days of accelerated conditions. They attributed this change to either water migration from the internal to the external aqueous phase or rupture of internal globules due to osmotic pressure.

In contrast, Moraes et al. [62] reported an increase in viscosity (7.89%) in formulations containing rutin succinate stored under accelerated conditions for 90 days. This finding, while inconsistent with most others, underscores the influence of extract composition, emulsion type, and formulation base on rheological stability outcomes.

##### Firmness and Adhesiveness

The increase in viscosity observed with the addition of GS extract to the control sample (time 0) aligns with the understanding that textural properties such as firmness and adhesiveness in cosmetic formulations are influenced by multiple factors, including the composition of the formulation. These properties are essential for evaluating the performance and user experience of cosmetic products.

Firmness refers to the resistance of a formulation to deformation under an applied force, reflecting its solidity and structural integrity. Adhesiveness measures the product’s ability to stick to the skin or other surfaces during application, thereby ensuring effective adherence and longer-lasting action.

Firmness influences how easily creams and lotions can be spread and manipulated, while adhesiveness is crucial for ensuring that the product remains on the skin to provide its intended effect over time. These parameters are also useful in selecting appropriate primary packaging; for instance, firmer formulations may require jars, whereas softer formulations are better-suited for tubes.

**Figure 3:**
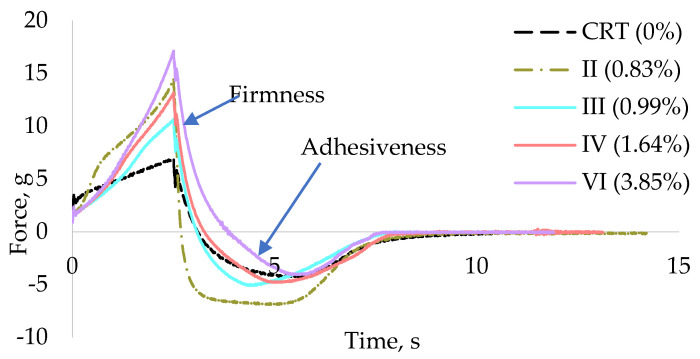
Texturometer curves for the samples CRT (0%), II (0.83%), III (0.99%), IV (1.64%), and VI (3.85% GS extract).

Texture-related properties were assessed to evaluate the influence of grape stem (GS) extract on firmness and adhesiveness. Figure 3 displays the texturometer curves of samples with different GS extract concentrations: CRT (0%), II (0.83%), III (0.99%), IV (1.64%), and VI (3.85%). Analysis of these curves allows the determination of firmness, corresponding to the highest positive peak, and adhesiveness, measured by the area under the negative portion of the curve.

The force–distance curves illustrate the mechanical resistance during compression, providing insights into texture-related properties such as firmness and adhesiveness. The shape and peak intensity of each curve reflect differences in internal structure associated with varying GS concentrations. Firmness is closely related to the hardness of a formulation, a key attribute in cosmetic products due to their typically semi-solid or non-liquid nature, which naturally resists applied force. However, excessive firmness may negatively affect the spreadability of the product on the skin, reducing user comfort and ease of application. Studies by Tai et al. [70] and Yarovaya et al. [63] reported firmness values for various commercial cosmetic products, including shampoos, hair conditioners, and sunscreens, ranging from 11.20 g to 15.90 g. Similarly, Huynh et al. [71] evaluated a broader spectrum of emulsions, reporting firmness values between 4.2 g and 40.5 g.

**Figure 4:**
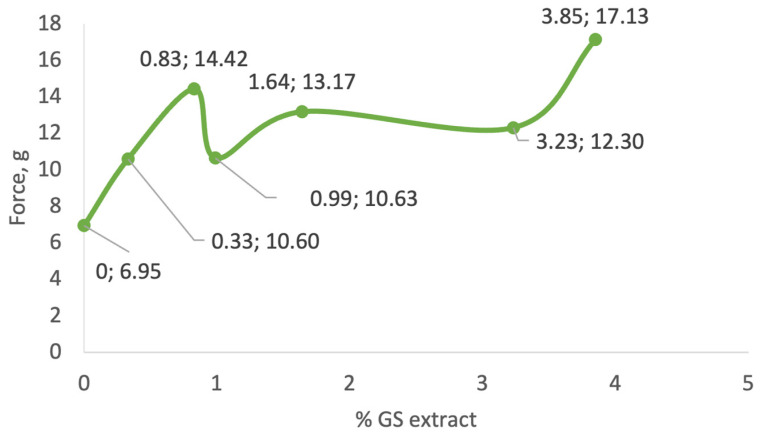
Firmness values of the formulations, as determined by texture analysis. The general increase in firmness values observed in the developed formulations can be attributed to the incorporation of GS extract, which contains solid components. These components likely contribute to the structural reinforcement of the emulsion, resulting in increased firmness. However, the relationship between GS extract concentration and firmness is not linear. This inconsistency may stem from disruptions in the balance between GS extract content and other formulation ingredients, such as thickeners or emulsifiers, which could modulate the final texture. The results indicate the force required to deform the samples, revealing the structural integrity of the gels. An increase in GS extract concentration generally correlates with enhanced firmness, suggesting reinforcement of the gel network by bioactive compounds.

As illustrated in Figure 4, the incorporation of GS extract into the cream base generally led to an increase in formulation firmness. Samples I (0.33% GS extract) and III (0.99%) displayed firmness values of 10.60 g and 10.63 g, respectively, which were moderately higher than the control sample (CRT, 6.95 g). In contrast, sample VI (3.85% GS extract) exhibited the highest firmness value, 17.13 g, indicating a more rigid and structurally reinforced formulation.

Interestingly, the findings from this study partially contrast with previous reports. For example, Yarovaya et al. [63] found no significant changes in firmness upon incorporating 3% grape seed extract into a sunscreen formulation. Similarly, Pinto et al. [72] observed a decrease in firmness (from 0.554 to 0.288 N) as the concentration of natural phenolic-rich extracts increased, suggesting that extract composition and base formulation play key roles in determining final texture.

##### Adhesiveness

Adhesiveness refers to the formulation’s ability to adhere to the skin, a property crucial for ensuring effective delivery and prolonged residence of cosmetic actives. Ideally, a formulation should demonstrate balanced adhesiveness: low enough to avoid a sticky, unpleasant sensation, yet high enough to ensure proper adherence and efficacy. Excessively low adhesiveness may reduce skin contact time and efficacy, while overly high values can impair spreadability and lead to user discomfort.

Huynh et al. [71] reported adhesiveness values for various emulsions ranging between −6.00 and −23.6 g·s, while Yarovaya et al. [63] observed average values around −22.5 g·s in commercial sunscreens.

**Figure 5:**
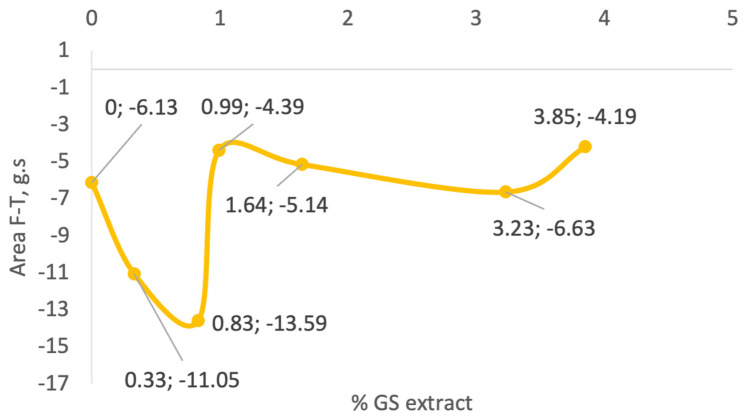
Adhesiveness of the formulations, as assessed by the work required to overcome attractive forces between the probe and sample.

As illustrated in Figure 5, there is no clear correlation between GS extract concentration and adhesiveness across the tested samples. Initially, as the GS extract concentration increases to 0.83% (sample II), adhesiveness increases markedly from −6.13 g·s (control) to −13.59 g·s. However, with further increases in GS concentration, adhesiveness values decline, reaching −4.49 g·s in sample III (0.99%), and slightly increase again to −6.63 g·s in sample V (3.23%).

Samples III (−4.34 g·s), IV (−5.14 g·s), and V (−6.63 g·s) show adhesiveness values comparable to the control, whereas samples I (0.33%) and II (0.83%) exhibit significantly higher adhesiveness (−11.05 and −13.59 g·s, respectively). This variability suggests that other formulation components or interactions with the GS extract influence adhesive behaviour more than concentration alone. This parameter reflects the stickiness of the gel. Intermediate GS concentrations show the highest adhesiveness, while both lower and higher concentrations exhibit reduced adhesion, possibly due to changes in matrix cohesion or moisture retention.

Pinto et al. [72] also reported a decrease in adhesiveness values (from −1.48 to −0.707 N·mm) as the concentration of phenolic-rich natural extracts increased in their formulations. Similarly, Yarovaya et al. [63] observed a slight reduction in adhesiveness, from −13.2 to −12.1 g·s, after incorporating 3% grape seed extract into a cosmetic base. In the present study, sample V (3.23% GS extract) exhibited an adhesiveness value (−6.63 g·s) like that of the control sample (−6.13 g·s). This effect is likely attributed to the astringent nature of polyphenols, which are known to remove excess surface oil and induce a tightening sensation on the skin, contrasting with the smooth or sticky feeling often associated with higher adhesiveness.

Textural properties were assessed only at the initial time point (time 0) to provide a baseline characterization. According to Jimtaisong et al. [73], noticeable texture changes are expected when viscosity variations exceed 30%. Accordingly, samples IV (1.64%) and VI (3.85%) likely experienced such changes, with viscosity increases of 37% and 58% determined, respectively, during storage.

The initial analysis revealed a consistent trend in the behaviour of formulations containing GS extract, up to a concentration of approximately 1%. Parameters, including viscosity, consistency index, firmness, adhesiveness, and flow-behaviour index, showed progressive increases with GS extract incorporation. Beyond this critical concentration, however, the relationship between extract content and physicochemical behaviour became inconsistent. This suggests a disruption in the formulation balance, possibly due to excessive dilution of the thickening agent or interactions among ingredients.

Additionally, spreadability and thixotropic behaviour were key factors influencing viscosity reduction during application. Samples with higher GS extract concentrations, particularly those containing 3.85% and 6.25%, tended to exhibit increased spreadability. However, this characteristic may also result in greater runoff and reduced adhesion to the skin, potentially affecting the delivery and absorption of bioactive compounds. Although adhesiveness was not directly measured for all highly concentrated samples, sample VI (3.85%) displayed the lowest adhesiveness among those tested, reinforcing the idea of a trade-off between spreadability and skin adherence.

Conversely, samples with greater adhesiveness—such as sample II (0.83%), with an adhesiveness value of −13.59 g·s—also exhibited higher flow-behaviour index values (0.2603) and lower thixotropy (1656 Pa·s). These results suggest that higher adhesion can hinder spreadability, emphasizing the importance of optimizing both properties to ensure desirable product performance.

Despite variations, all formulations remained within acceptable rheological and textural ranges for cosmetic emulsions, retaining shear-thinning behaviour that facilitates application and subsequent recovery of consistency on the skin.

Following accelerated storage (time 3), a comparative analysis of all rheological parameters identified sample IV (1.64% GS extract) as the least stable, with the most substantial changes observed (ranging from 37% to 65%) in all variables except thixotropy. In contrast, sample III (0.99%) was the most stable, exhibiting only a 9% decrease in consistency index and increases of 23% and 21% in flow-behaviour index and viscosity, respectively.

#### 3.1.5. Quantification of Phenolic Compounds and Long-Term Assessment

Quantification of total phenolic content (TPC) and flavonoid content (FC) was conducted after incorporating GS extract into the formulations. As GS extract is rich in phenolic compounds, particularly flavonoids, evaluating their retention in the final product is essential for understanding both chemical stability and potential bioactivity. Results are presented in Table 8.

In a previous study, the GS extract was characterized in terms of total TC, ODC, and FC, with values of 78.55 ± 1.67 mg GAE/g dry weight for TPC and 66.77 ± 1.96 mg CAT/g dry weight for FC [22].

Cosmetic formulations enriched with different concentrations of grape stem (GS) extract exhibited marked variations in total phenolic content (TPC) and flavonoid content (FC). Overall, increases in TPC and FC were observed as the concentration of GS extract increased, with values ranging from 9.44 ± 0.79 to 232.65 ± 14.11 mg GAE/100 g cream (TPC) and from 44.04 ± 4.07 to 274.60 ± 6.68 mg CAT/100 g cream (FC). These results are consistent with findings by Censi et al. [74] and Namngam et al. [75], who also reported increases in phenolic content following the incorporation of natural extracts into cosmetic emulsions.

However, the increase in phenolic content was not strictly proportional to the amount of extract added. The most notable rise in TPC occurred at 0.83% GS extract, whereas a significant increase in FC was only observed from 3.23% onward. This discrepancy may reflect the complex chemical composition of the GS extract, as individual compounds vary in solubility, stability, and incorporation efficiency within an emulsion matrix [74,75,76].

Over time, most formulations showed a general decline in TPC following the first month of accelerated storage. Notably, formulation IV (1.64%) retained nearly all of its initial TPC after one month (from 44.40 to 43.54 mg GAE/100 g cream) and, along with formulation III (0.99%), demonstrated the highest overall stability, with only a 16% reduction after three months. Formulations VI (3.85%) and I (0.33%) showed decreases of 20% and 29%, respectively. Between the first and third months, most samples remained relatively stable, except for formulation VII (6.25%), which showed a 44% decrease, and formulation II (0.83%), which experienced the largest loss (47%).

Flavonoid stability followed a slightly different trend. Formulations with lower extract concentrations generally exhibited greater FC retention. Formulations I (0.33%), III (0.99%), and V (3.23%) maintained their flavonoid content throughout the three-month period without statistically significant changes. In contrast, formulation IV (1.64%) retained its FC only through the first month, with an 18% decrease by the end of the study. The most concentrated formulation (VII, 6.25%) showed the lowest stability, with a 53% loss in flavonoid content over three months.

The decreases in phenolic and flavonoid contents over time can be attributed to chemical reactions occurring within the formulation. Processes such as oxidation, hydrolysis, and transesterification may lead to the degradation of these compounds, resulting in reduced concentrations. Furthermore, the formation of new chemical species during these reactions may contribute to the observed decline, as these newly formed compounds may not be detected by the analytical methods typically used to quantify total phenolic and flavonoid contents.

These findings are consistent with previous studies. For example, Salem et al. [48] reported a decrease in total phenolic content (TPC) in creams containing grape seed extract at concentrations ranging from 0% to 5% over four months, with the most pronounced reduction observed in the 5% sample. Notably, significant differences were detected as early as the first month at 50 °C. Similarly, Rodrigues et al. [49] observed a reduction in TPC, from 4192.95 ± 50.25 to 2918.71 ± 90.42 µg GAE/mL, in creams with natural extracts after 180 days of accelerated storage. Maisuthisakul et al. [77] also reported a time-dependent decline in TPC in formulations containing natural extracts under accelerated conditions. Esparza et al. [78] studied grape stem extract stability over six months, observing a TPC reduction from 0.50 to 0.33 mmol GAE/g extract.

#### 3.1.6. Antioxidant, Antiaging, and Depigmenting Activities Evaluation

##### Antioxidant Activity

The antioxidant activity of the formulations was evaluated using ABTS and FRAP assays, which are widely applied to assess the ability of compounds to neutralize free radicals and reduce ferric ions, respectively. The relevance of phenolic compounds in these mechanisms is well-documented in the literature [77,78,79,80].

As presented in Table 9, antioxidant activity increased with higher concentrations of GS extract, with ABTS values ranging from 0.01 ± 0.00 to 4.14 ± 0.33 mmol TROLOX/100 g cream, and FRAP values from 0.02 ± 0.00 to 3.10 ± 0.02 mmol TROLOX/100 g cream. This increase, however, did not follow a strictly linear pattern. For instance, a marked rise in ABTS activity was only observed from the 0.83% concentration, whereas the FRAP results showed a more progressive increase beginning at 0.33%.

These differences likely reflect the distinct oxidative mechanisms evaluated by each assay. While ABTS is sensitive to radical-scavenging activity, FRAP measures the ability of antioxidants to donate electrons and reduce Fe^3+^ to Fe^2+^. Therefore, the performance of specific phenolic compounds may vary depending on their structure, reactivity, and interaction with each system.

The non-linear trend may also be associated with the complex composition of the GS extract, which includes a diverse array of phenolics, such as flavonoids and phenolic acids. These compounds differ in solubility, antioxidant potential, and stability within the cream matrix [81,82], which can influence their contribution to its overall activity.

The observed antioxidant activity correlated with the extract concentration, providing an indirect reflection of phenolic content across the tested samples. However, values were not normalized per mg of total phenolics; this will be addressed in future studies.

The literature reports similar observations. Censi et al. [74] described a non-proportional increase in antioxidant activity in emulsions with açaí extract, while Bernatoniene et al. [83] found no significant change in DPPH radical-scavenging activity until calendula extract exceeded a certain concentration threshold, reinforcing the idea that efficacy may depend on reaching critical compound levels.

Antioxidant stability under accelerated conditions was also assessed. Most formulations showed a decline in activity over time, particularly between the first and third months. Among them, samples III (0.99%) and IV (1.64%) demonstrated the greatest stability, maintaining their antioxidant capacity during the first month and exhibiting overall decreases of 58% and 61% (ABTS), respectively, over three months. Samples with lower extract concentrations, such as I (0.33%) and II (0.83%), were less stable, with reductions reaching up to 80%.

A similar trend was observed in the FRAP assay. Samples III and IV again showed the highest retention of ferric-reducing activity, with losses of 30% and 32%, respectively. Formulations containing 3.23% and 6.25% GS extract followed, with 39% and 40% reductions. The formulation with the lowest extract content (0.33%) showed the greatest loss in ferric-reducing capacity over time.

Similar findings were reported by Salem et al. [48], who observed a 40% decrease in antioxidant activity (assessed via DPPH) in creams containing 5% grape seed extract (Marselan variety) stored at 50 °C for four months. Esparza et al. [78] also investigated antioxidant activity using the DPPH assay in grape stem extracts and reported a decrease from 0.45 to 0.29 mmol TROLOX/g extract (36%) after six months under accelerated conditions.

The incorporation of grape stem (GS) extract into cosmetic formulations significantly enhanced their antioxidant activity. These results, consistent with findings from the literature, highlight the important role of phenolic compounds in improving the functional and protective potential of cosmetic products. Antioxidants are crucial in mitigating oxidative stress, a major contributor to skin aging. The accumulation of reactive oxygen species (ROS) leads to cellular damage and senescence, accelerating cutaneous aging processes [83,84]. Therefore, phenolic compounds, known for their antioxidant properties, offer antiaging benefits by neutralizing free radicals and preventing oxidative degradation. Antioxidant evaluation thus serves as a relevant proxy for predicting the antiaging efficacy of cosmetic formulations.

Nonetheless, the antioxidant capacity of the formulations declined during the stability study, as reflected in both the ABTS and FRAP results. This decline correlates with the previously observed decrease in total phenolic content (TPC). As phenolic levels decreased, so too did antioxidant performance, confirming the central role of these compounds. Furthermore, interactions between phenolic compounds and other formulation components, such as emulsifiers, preservatives, or stabilizers, may influence the chemical stability and functional effectiveness of the compounds, contributing to the observed loss of antioxidant activity over time. Although the DPPH and FRAP assays are not fully representative of cutaneous antioxidant mechanisms, they remain widely accepted as sensitive chemical models used for assessing oxidative stability in formulations during storage. These assays provide a useful preliminary indication of the retention of antioxidant potential within the cream matrix over time. However, it is important to consider that both methods can be influenced by the presence of emulsifiers and lipid components in the cosmetic base. Such ingredients may interfere with the release, solubility, or reactivity of phenolic compounds, potentially affecting the extraction efficiency and sensitivity of the assays. Despite employing hydroalcoholic solvents to facilitate phenolic recovery from the cream, the complexity of the emulsion system may still limit complete extraction or alter the kinetics of the redox reactions measured. Therefore, while the observed antioxidant activity trends generally correlate with the concentration of grape stem extract and its known phytochemical composition, these results should be interpreted with caution due to potential matrix effects.

In this study, total phenolic content was measured as an indicator of bioactive compound presence, yet a detailed statistical correlation between phenolic concentration and antioxidant or antimicrobial activity over time was not performed. Nonetheless, the observed trends suggest a positive relationship between phenolic levels and antioxidant capacity, supporting the role of these compounds in maintaining the cream’s bioactivity.

Future research will aim to perform more rigorous correlation and dose–response analyses, alongside molecular-level investigations, to elucidate the mechanisms underlying the antioxidant and antimicrobial effects of the grape stem extract within the cosmetic matrix. Additionally, studies using biologically relevant models or cell-based assays are warranted to validate antioxidant functionality under conditions that better mimic topical application.

##### Antiaging and Depigmenting Activities

The antiaging efficacy of the cosmetic formulations was investigated through their ability to inhibit elastase activity. Elastase, a zinc-dependent enzyme also known as human macrophage metalloelastase, plays a key role in the degradation of elastin—an essential protein for maintaining skin elasticity and firmness. Its increased activity in aged or photoaged skin contributes to the appearance of wrinkles and loss of dermal structure [85,86]. Therefore, elastase inhibition is widely recognized as a relevant strategy in the development of antiaging skincare products.

In addition, the potential depigmenting effect of the formulations was evaluated via tyrosinase inhibition. Tyrosinase is a copper-containing enzyme that catalyzes the rate-limiting step in melanogenesis, namely the oxidation of tyrosine to L-DOPA. Enhanced tyrosinase activity has been associated with age-related hyperpigmentation, including dark spots and uneven skin tone. As such, reducing this enzymatic activity is a valuable approach in the prevention and treatment of skin discoloration [87,88,89,90].

The results revealed that elastase inhibition ranged from 40.05 ± 1.67% to 52.83 ± 2.50%, with the control formulation (0% GS extract) showing a baseline inhibitory effect of 22.98 ± 9.13% (Table 10). The lack of a direct correlation between extract concentration and enzyme inhibition suggests the presence of a threshold beyond which no significant enhancement occurs. For instance, low- and high-concentration samples (e.g., 0.33% and 6.25%) displayed similar inhibitory levels, indicating a possible saturation effect within the formulation.

Regarding tyrosinase inhibition, only formulations containing higher concentrations of GS extract—specifically, samples V (3.23%), VI (3.85%), and VII (6.25%)—retained appreciable inhibitory capacity, with values ranging from 11.64 ± 6.50% to 29.44 ± 4.02%. Lower-concentration formulations presented minimal activity, suggesting that the active compounds responsible for tyrosinase inhibition may require higher concentrations to remain effective within the cream matrix.

These findings are consistent with the previous literature. For example, Hong et al. [91] reported that a 5% green tea extract formulation exhibited 24.79% elastase inhibition and 35.38% tyrosinase inhibition. Similarly, Anggraini et al. [68] observed improved skin elasticity with a 10% Rhus javanica extract cream, further supporting the role of plant-based emulsions in promoting antiaging benefits.

The observed reduction in tyrosinase activity across formulations aligns with earlier reports on the instability of certain phenolic compounds in cosmetic systems. Taofiq et al. [92], for instance, described tyrosinase activity losses of up to 95% following the incorporation of mushroom-derived extracts into emulsions. In contrast, the present formulations retained a greater portion of their initial activity, with losses varying between 36% and 74%, depending on the concentration.

Stability tests under accelerated conditions indicated that formulations with lower extract concentrations generally preserved their elastase inhibitory properties more effectively over time. Sample II (0.83%) showed no significant variation over the three-month period, and samples I (0.33%) and IV (1.64%) also demonstrated high stability, with activity reductions of 5% and 20%, respectively. Conversely, formulations with higher concentrations (i.e., samples V, VI, and VII) were more susceptible to activity loss during storage.

As for tyrosinase inhibition, only sample V (3.23%) retained measurable activity after three months, suggesting that this concentration may represent an optimal balance between effectiveness and stability in the formulation context.

## 4. Conclusions

This study successfully incorporated grape stem (GS) extract into a cosmetic cream formulation, resulting in increases in total phenolic and flavonoid contents. However, compared to the isolated extract, a reduction of approximately 52% in total phenolic content was observed in the formulations, likely due to the limited solubility of certain phenolic compounds in the cream matrix. In contrast, flavonoid content remained largely stable, suggesting that flavonoids are more soluble and better retained in the cream base.

Despite the successful incorporation of GS extract, some physical and chemical changes were noted, specifically, colour, odour, pH, viscosity, and spreadability. Nonetheless, these changes remained within acceptable limits. Colour changes (browning) and odour shifts (towards a woody scent) were characteristic of the GS extract and do not indicate product degradation. The pH, although reduced with increasing GS concentration, remained within the physiologically compatible range for skin (4.00–4.50).

The rheological and textural properties exhibited clear patterns. Viscosity increased with GS extract incorporation, peaking between 0.83% and 1.64%, then fluctuating irregularly at higher concentrations. This behaviour was also observed in parameters such as the consistency index (K) and firmness. Despite these fluctuations, all formulations maintained the structural characteristics expected of cosmetic products.

Spreadability tended to decrease with higher GS extract concentrations, particularly within the 0.83–1.64% range. This was reflected in increases in the flow-behaviour index, thixotropy, and adhesiveness. However, all formulations retained their desirable shear-thinning behaviour, allowing them to become more fluid under stress (e.g., during application) and recover viscosity afterward—an important attribute for topical cosmetic use.

In terms of biological activity, the GS extract conferred antioxidant and antiaging benefits upon the cream. However, antioxidant capacity was reduced by approximately 50% in the ABTS assay and 25% in the FRAP assay, compared to the pure extract, likely due to the limited solubility or stability of certain phenolic compounds. Elastase inhibition—a marker of antiaging potential—was largely maintained, while tyrosinase inhibition (linked to depigmenting activity) was retained only in formulations with higher GS extract concentrations. This suggests the bioactive compounds responsible for tyrosinase inhibition may not be adequately solubilized or stabilized in lower concentrations.

Three-month accelerated stability studies (at 40 ± 2 °C and 75 ± 5% RH) revealed that the most concentrated formulations (≥3.23% GS extract) were the least stable, showing increased lumpiness (due to coalescence) and darker coloration. No clear trends in viscosity or consistency were observed in these samples, likely due to physical destabilization. Nevertheless, formulations such as samples II, III, and IV (0.83–1.64% GS extract) demonstrated greater stability in key parameters including consistency index, viscosity, thixotropy, and flow-behaviour index. Sample IV was particularly stable in terms of flow-behaviour index.

Importantly, all formulations maintained safe pH values and remained microbiologically stable throughout the testing period, with microbial counts consistently below 100 CFU/g.

With respect to phenolic retention, samples III and IV were most effective in maintaining total phenolic content, while samples I to IV best preserved flavonoid content. Biologically, samples II and III retained their antioxidant properties (ABTS), while samples III and IV preserved FRAP activity. Sample II also sustained elastase inhibition capacity over time.

In summary, the most stable and bioactive formulations were those containing GS extract concentrations between 0.83% and 1.64%. Conversely, higher concentrations tended to compromise formulation stability and performance.

These findings highlight the potential of grape stem extract as a valuable ingredient for cosmetic formulations, offering antioxidant and antiaging benefits while remaining stable over time. Future studies should aim to investigate a broader range of GS extract concentrations, particularly those between 0.5% and 1.5%, to determine the formulation with optimal stability and efficacy. Exploring encapsulation methods may further improve the stability of the extract and enable controlled release of active compounds. Evaluating the incorporation of GS extract into different cream bases could help identify the formulation environment most suitable for preserving bioactivity. Additionally, conducting long-term in vivo studies is essential to assess the cream’s effects on parameters such as skin elasticity, hydration, and wrinkle reduction, ultimately ensuring its effectiveness and supporting its commercial viability. It is important to note that while the same base cream was used across all formulations, including the control (CRT), this matrix contained a commercial preservative system, but no added antioxidants or phenolic ingredients. Although this provided a consistent comparative baseline, the lack of a control formulation with ethanol alone may slightly limit the interpretation of antioxidant and microbiological data, as ethanol—although mostly removed—could have had residual effects. Future studies will include additional controls (e.g., base cream + ethanol) and replicate batches to strengthen statistical power and mechanistic interpretation.

## 5. Future Perspectives and Translational Relevance

Although the present study focuses mainly on the formulation development and physicochemical stability of creams enriched with grape stem extract, it is important to acknowledge that the translational relevance of these findings requires further validation under conditions which are more biologically representative. The current work establishes a foundation for the potential use of these formulations as antioxidant and bioactive skincare products; however, additional investigations are necessary to fully assess their efficacy and safety in realistic contexts.

Future research will involve in vitro permeation and penetration studies using human or reconstructed skin models to evaluate the bioavailability and the delivery of phenolic compounds from the cream matrix to the target skin layers. Such studies will provide critical insight into the ability of the active compounds to reach, and exert their effects, within the skin, which is essential to confirm the functional benefits suggested by the in vitro antioxidant assays presented here.

Moreover, cytotoxicity and irritation assessments in relevant skin models, as well as controlled clinical or consumer sensory trials, will be pursued to establish the safety profile, dermatological compatibility, and user acceptability of the formulations. These steps are fundamental in ensuring that the product not only maintains its bioactivity but is also safe and pleasant to use, supporting potential commercialization.

The intended use of these formulations is primarily topical application in the cosmetic and dermocosmetic market, targeting consumers who value natural, sustainable ingredients with scientifically supported antioxidant and skin-protective properties. Incorporating by-products such as grape stems into high-value cosmetic products aligns with current trends in sustainability and the circular economy, which also adds value from the environmental and marketing perspectives.

In summary, while this study provides important initial data on formulation and stability, the progression towards clinical validation and regulatory approval will require a multidisciplinary approach. This will include mechanistic studies at the molecular level, in vitro and in vivo biological evaluations, and comprehensive safety and efficacy assessments, ultimately aiming to deliver effective and consumer-friendly products that contribute positively to skin health.

## Figures and Tables

**Table 1:** Concentration of GS extract in water gel cream base.

Sample	g GS Powder/30 g Cream Base	% GS
CRT	0.000	0
I	0.100	0.33
II	0.250	0.83
III	0.300	0.99
IV	0.500	1.64
V	1.000	3.23
VI	1.200	3.85
VII	2.000	6.25

**Table 2:** Water gel cream base composition.

Function	Ingredient	%
Thickening, stabilizer, texturizing agent	Polyacrylamide, C13-14 Isoparaffin, Laureth-7	91.5
Preservative	Methylchloroisothiazolinone, methylisothiazolinone	3.4
Fragrance	Parfum	3.5
Solvent	Water	1.6

**Table 3:** Correspondence, based on the Q rule (Q = 2), between the storage times of samples under accelerated stability conditions and normal conditions.

Storage Time (Months)—Accelerated Conditions	Storage Time (Months)—Normal Conditions
1	4
2	8
3	12

**Table 4:** Organoleptic characteristics of the cosmetic formulations immediately after preparation.

Sample (% Extract)	Colour	Appearance	Odor
CRT (0%)	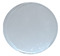	Glossy and shiny Creamy and soft texture	Parfum
I (0.33%)	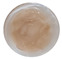	Glossy and shiny Creamy and soft texture	Parfum
II (0.83%)	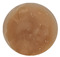	Glossy and shiny Creamy and soft texture	Parfum Woody
III (0.99%)	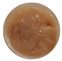	Glossy and shiny Creamy and soft texture	Parfum Woody
IV (1.64%)	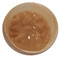	Glossy and shiny Creamy and soft texture	Parfum Woody
V (3.23%)	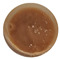	Glossy and shiny Thinner texture	Parfum Woody
VI (3.85%)	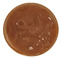	Glossy and shiny Thinner texture	Woody
VII (6.25%)	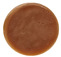	Glossy and shiny Thinner texture	Woody

**Table 5:** Colour change over a period of 3 months.

	Months in a Stability Chamber			
Sample (% Extract)	0	1	2	3
CRT (0%)	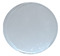	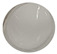	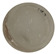	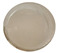
I (0.33%)	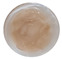	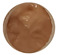	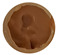	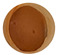
II (0.83%)	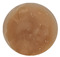	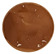	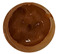	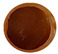
III (0.99%)	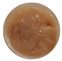	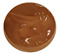	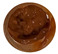	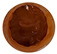
IV (1.64%)	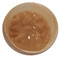	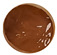	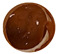	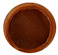
V (3.23%)	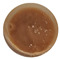	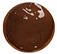	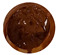	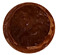
VI (3.85%)	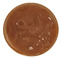	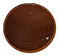	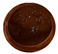	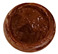
VII (6.25%)	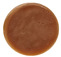	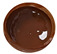	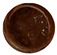	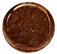

**Table 6:** Variations in pH of developed samples during storage in stability chamber.

	Months in a Stability Chamber				
Sample (% GS Extract)	0	1	2	3	Variance
CRT (0%)	6.34	6.08	6.05	5.99	0.018
I (0.33%)	4.50	4.49	4.69	4.69	0.010
II (0.83%)	4.21	4.20	4.32	4.24	0.002
III (0.99%)	4.18	4.16	4.32	4.18	0.004
IV (1.64%)	4.01	4.08	4.21	4.05	0.006
V (3.23%)	4.02	3.99	4.05	3.92	0.002
VI (3.85%)	3.97	3.95	4.11	3.88	0.007
VII (6.25%)	3.93	3.94	3.96	3.84	0.002

**Table 7:** Variations in the flow-behaviour index (n) and consistency index (K) of the cosmetic formulations containing different concentrations of grape stem (GS) extract, measured at time 0 (initial) and time 3 (after 3 months of storage under accelerated conditions).

	n Flow-Behaviour Index		K Consistency Index	
Sample (% GS Extract)	Time 0	Time 3	Time 0	Time 3
CRT (0%)	0.1878	0.2362	46.697	97.904
I (0.33%)	0.2225	0.2851	137.170	122.020
II (0.83%)	0.2603	0.3030	150.410	78.248
III (0.99%)	0.2240	0.2760	161.190	146.220
IV (1.64%)	0.2237	0.3276	139.810	49.434
V (3.23%)	0.2365	0.2873	94.043	78.453
VI (3.85%)	0.2208	0.2665	90.967	106.270
VII (6.25%)	0.1858	0.2826	129.480	88.792

The observed changes in rheological properties over time are significant indicators of the evolving behaviour of these cosmetic formulations. The flow-behaviour index values increased from time 0 to time 3 across all samples, indicating a reduction in shear-thinning behaviour. This reduction was particularly noticeable in samples IV and VII (1.64% and 6.25% GS extract, respectively). Conversely, the consistency index values decreased, except for samples CRT (0% GS extract) and VI (3.85% GS extract). Consequently, the force levels required to commence flow in the samples decreased compared to the initial time, with the most significant differences observed in samples II (0.83% GS extract) and IV (1.64% GS extract).

**Table 8:** Total phenolic and flavonoid contents over time.

	Total Phenolics (mg GAE/100 g Cream) *			Flavonoids (mg CAT/100 g Cream) **		
	Months in a Stability Chamber			Months in a Stability Chamber		
Sample (% GS Extract)	0	1	3	0	1	3
CRT (0%)	9.44 ± 0.79 ^aE^	5.72 ± 0.40 ^b^	5.39 ± 0.52 ^bF^	44.04 ± 4.07 ^aD^	50.06 ± 3.22 ^a^	24.65 ± 1.41 ^bD^
I (0.33%)	16.35 ± 1.91 ^aE^	11.04 ± 1.22 ^b^	11.58 ± 0.18 ^bEF^	45.10 ± 3.71 ^aD^	53.58 ± 5.20 ^a^	47.36 ± 2.41 ^aC^
II (0.83%)	34.30 ± 2.99 ^aD^	15.96 ± 1.01 ^b^	18.17 ± 0.12 ^bE^	62.88 ± 0.71 ^aD^	56.91 ± 2.82 ^b^	46.58 ± 0.98 ^cC^
III (0.99%)	37.50 ± 3.39 ^aD^	29.51 ± 1.15 ^b^	31.65 ± 0.33 ^bD^	66.38 ± 3.32 ^aD^	63.37 ± 4.34 ^a^	61.08 ± 1.17 ^aC^
IV (1.64%)	44.40 ± 0.97 ^aD^	43.54 ± 2.71 ^ab^	37.42 ± 3.80 ^bD^	64.94 ± 3.89 ^aD^	67.76 ± 2.50 ^a^	53.34 ± 1.97 ^bC^
V (3.23%)	102.38 ± 3.92 ^aC^	64.85 ± 1.88 ^b^	65.45 ± 4.84 ^bC^	130.93 ± 14.06 ^aC^	117.05 ± 4.21 ^a^	107.78 ± 12.89 ^aB^
VI (3.85%)	127.70 ± 6.29 ^aB^	97.95 ± 1.21 ^b^	101.70 ± 3.22 ^bB^	162.48 ± 17.32 ^aB^	102.87 ± 2.70 ^b^	117.36 ± 13.91 ^bAB^
VII (6.25%)	232.65 ± 14.11 ^aA^	175.75 ± 3.1 ^b^	129.37 ± 2.56 ^cA^	274.60 ± 6.68 ^aA^	211.09 ± 13.45 ^b^	128.91 ± 5.08 ^cA^

Results are presented as mean ± standard deviation (*n* = 3). * Results are presented a mean ± standard deviation (*n* = 3) and are expressed in mg GAE/g dw (dry weight). ** Results are presented as mean ± standard deviation (*n* = 3) and are expressed in mg CAT/g dw. Significant differences between samples in the same row are denoted by different lowercase letters, while significant differences between samples in the same column are indicated by different capital letters (*p* < 0.05).

**Table 9:** Antioxidant activity by ABTS and FRAP assays.

	ABTS *			FRAP *		
	Months in a Stability Chamber			Months in a Stability Chamber		
Sample (% GS Extract)	0	1	3	0	1	3
CRT (0%)	0.01 ± 0 ^bE^	0.03 ± 0 ^a^	0 ± 0 ^cE^	0.02 ± 0 ^bF^	0.05 ± 0.01 ^a^	0.02 ± 0 ^bF^
I (0.33%)	0.17 ± 0.02 ^aDE^	0.13 ± 0.01 ^b^	0.03 ± 0 ^cDE^	0.22 ± 0.02 ^aE^	0.24 ± 0.02 ^a^	0.06 ± 0 ^bF^
II (0.83%)	0.49 ± 0.01 ^aCD^	0.41 ± 0.03 ^b^	0.10 ± 0 ^cD^	0.55 ± 0.05 ^aD^	0.46 ± 0.01 ^b^	0.20 ± 0 ^cE^
III (0.99%)	0.50 ± 0.05 ^aCD^	0.51 ± 0.05 ^a^	0.21 ± 0.01 ^bC^	0.52 ± 0.03 ^aD^	0.43 ± 0.02 ^b^	0.36 ± 0.01 ^cD^
IV (1.64%)	0.66 ± 0.05 ^aC^	0.79 ± 0.07 ^a^	0.26 ± 0.02 ^bC^	0.71 ± 0.03 ^aC^	0.62 ± 0.02 ^b^	0.48 ± 0.02 ^cC^
V (3.23%)	1.83 ± 0.15 ^aB^	1.46 ± 0.02 ^b^	0.72 ± 0.02 ^cB^	1.57 ± 0.09 ^aB^	1.32 ± 0.05 ^b^	0.96 ± 0.04 ^cB^
VII (6.25%)	4.14 ± 0.33 ^aA^	3.51 ± 0.11 ^b^	1.50 ± 0.05 ^cA^	3.10 ± 0.02 ^aA^	2.56 ± 0.04 ^b^	1.86 ± 0.02 ^cA^

Results are presented as mean ± standard deviation (*n* = 3) and are expressed in mmol TROLOX/100 g cream. * Results are presented as mean ± standard deviation (*n* = 3) and are expressed in mmol TROLOX/g dw. Significant differences between samples in the same row are denoted by different lowercase letters, while significant differences between samples in the same column are indicated by different capital letters (*p* < 0.05).

**Table 10:** Evaluation of elastase and tyrosinase inhibition.

	Elastase Inhibition Assay			Tyrosinase Inhibition Assay		
	Months in a Stability Chamber			Months in a Stability Chamber		
Sample (% GS Extract)	0	1	3	0	1	3
CRT (0%)	22.98 ± 9.13 ^aC^	31.45 ± 3.41 ^a^	19.40 ± 4.24 ^aC^	0 * ^CD^	-	-
I (0.33%)	53.22 ± 0.89 ^bA^	56.96 ± 0.51 ^a^	50.22 ± 0.45 ^cA^	0 * ^E^	-	-
II (0.83%)	52.19 ± 3.57 ^aA^	53.62 ± 1.33 ^a^	52.47 ± 1.62 ^aA^	0 * ^DE^	-	-
III (0.99%)	47.61 ± 5.40 ^aAB^	44.33 ± 2.54 ^a^	29.62 ± 2.11 ^bB^	0 * ^C^	-	-
IV (1.64%)	40.05 ± 1.67 ^bB^	48.06 ± 2.67 ^a^	32.06 ± 1.56 ^cB^	0 * ^CD^	-	-
V (3.23%)	5.40 ± 1.01 ^aD^	4.72 ± 5.53 ^a^	9.39 ± 1.19 ^aD^	11.64 ± 6.50 ^aB^	10.68 ± 1.90 ^a^	10.04 ± 3.10 ^aA^
VI (3.85%)	44.15 ± 1.31 ^aAB^	0 * ^c^	7.00 ± 7.11 ^bD^	22.69 ± 9.25 ^aAB^	13.99 ± 2.62 ^a^	0 * ^bB^
VII (6.25%)	52.83 ± 2.50 ^aA^	43.90 ± 2.94 ^b^	12.74 ± 2.74 ^cCD^	29.44 ± 4.02 ^aA^	21.36 ± 1.74 ^a^	0 * ^bB^

Results are presented as mean ± standard deviation (*n* = 3) and are expressed as % inhibition. The values described as time zero refer to the values obtained during the full chemical characterization of the extract in a previous study [22]. Significant differences between samples in the same row are denoted by different lowercase letters, while significant differences between samples in the same column are indicated by different capital letters (*p* < 0.05). * Not determined (below detection limit).

## Data Availability

The original contributions presented in this study are included in the article. Further inquiries can be directed to the corresponding author.
